# The association between Korean employed workers’ on-call work and health problems, injuries

**DOI:** 10.1186/s40557-018-0225-0

**Published:** 2018-03-20

**Authors:** Chulin Baek, Jae Bum Park, Kyungjong Lee, Jaehyuk Jung

**Affiliations:** 1 Department of Occupational and Environmental Medicine, Ajou University Hospital, Suwon, South Korea; 2 Department of Occupational and Environmental Medicine, Ajou University School of Medicine, Suwon, South Korea

**Keywords:** On-call work, Health problems, Injuries, Korean working conditions survey, Broad range of the job spectrum

## Abstract

**Background:**

On-call work is a form of work that requires the person to work at any time during the on-call period. Thus, on-call work is often regarded as one of the most severe stress factors. This study investigates the associations between on-call work and health problems, injuries.

**Methods:**

This study was based on the 3rd Korean Working Conditions Survey. Total of 29,246 employed workers who had been working for at least 1 year were included. Logistic regression analysis was performed to investigate the association between on-call work and health problems, injuries.

**Results:**

The odds ratios for on-call workers in terms of physical health problems, psychological health problems, and injuries were 1.33 (95% confidence interval [CI] 1.22-1.44), 1.31 (95% CI 1.08-1.60), and 2.76 (95% CI 2.26-3.37), respectively. Analysis of the detailed symptoms revealed odds ratios in on-call workers of 2.06 for hearing problems (95% CI 1.63-2.62); 1.71 for skin problems (95% CI 1.38-2.12); 1.22 for back pain (95% CI 1.08-1.38); 1.23 for muscular pains in upper limbs (95% CI 1.12-1.34); 1.27 for muscular pains in lower limbs (95% CI 1.15-1.40); 1.46 for headache, eye fatigue (95% CI 1.32-1.60); 1.37 for abdominal pain (95% CI 1.02-1.85); 1.43 for depression or anxiety disorders (95% CI 1.07-1.93); 1.36 for fatigue (95% CI 1.24-1.49); and 1.41 for insomnia and general sleep difficulties (95% CI 1.13-1.76).

**Conclusions:**

The present study found that on-call work results in an increased risk of health problems and injuries. This study is the result of analyses of broad range of the job spectrum in Korean employed workers; thus, future studies are necessary to determine the effects of on-call work in various job groups.

## 국문요약

**배경:**

대기근무는 언제든지 근무를 해야 하는 상황이 생길 수 있기 때문에 스트레스 상황이 빈번한 근무형태이다. 하지만 대기근무에 관한 선행 연구들은 특정 직종을 대상으로 한 연구가 대부분이다. 본 연구는 특정 직종이 아닌 전체 근로자들을 대상으로 대기근무와 건강문제 및 손상의 관련성을 파악하기 위하여 수행되었다.

**방법:**

본 연구는 2011 년 시행한 제 3차 근로환경조사 자료를 활용 하였고, 대상자는 1 년이상 근무한 임금 근로자 29,246 명으로 하였다. 대기근무와 건강문제 및 손상의 관련성을 확인하기 위해 로지스틱 회귀분석을 시행하였다.

**결과:**

전체 대상자의 9.3%인 2723 명이 대기근무를 하고 있는 것으로, 26,524 명 (90.7%)은 대기근무를 하지 않는 것으로 나타났다. 개인적 특성과 직업적 특성, 근무환경, 직무스트레스를 보정하여 분석한 결과 대기근무를 하는 경우 그렇지 않은 경우에 비해 신체적 증상 1.33 배 (95% CI 1.22-1.44), 정신적 증상 1.31 배 (95% CI 1.08-1.60), 손상 2.76 배 (95% CI 2.26-3.37) 높게 나타났다. 세부 증상에 대해서 분석한 결과 대기근무를 하는 경우 청력문제 2.06 배 (95% CI 1.63-2.62), 피부문제 1.71 배 (95% CI 1.38-2.12), 요통 1.22 배 (95% CI 1.08-1.38), 어깨, 목, 팔 등의 근육통 1.23 배 (95% CI 1.12-1.34), 하지의 근육통 1.27 배 (95% CI 1.15-1.40), 두통 및 눈의 피로 1.46 배 (95% CI 1.32-1.60), 복통 1.37 배 (95% CI 1.02-1.85), 우울 또는 불안장애 1.43 배 (95% CI 1.07-1.93), 전신피로 1.36 배 (95% CI 1.24-1.49), 불면증 및 수면장애 1.41 배 (95% CI 1.13-1.76) 높게 나타났다.

**결론:**

본 연구 결과, 대기근무가 건강문제 및 손상과 관련이 있다는 것을 확인 할 수 있었다. 추후 인과관계 파악을 위한 추가 연구가 진행되어야 하며 또한 다양한 직종에서도 대기근무와 관련된 연구가 필요할 것으로 보인다.

## Background

On-call work, by definition, is a form of work that requires the person to work at any time during the on-call period. Therefore, a person often has to work during both daytime and nighttime hours, leading to stressful situations for the worker [1]. Thus, on-call work is often regarded as one of the most severe stress factors [2–4]. This was reflected in a European working conditions survey (EWCS) performed to establish the foundation for creating a healthy work environment; on-call work assessment was first introduced in the 5th European EWCS performed in 2010. Similarly, in Korea, assessment of on-call work was first included the 2nd Korean Working Conditions Survey in 2010, which defined on-call work as “immediately providing work or service if contacted or called”. The present study also used this definition.

Previous studies have reported the various effects of on-call work on workers. The health-related effects of on-call work include stress-related symptoms such as exhaustion, irritation, sleep disorders, memory disorders, and headache [4]. A previous study on nurses reported that on-call work was associated with musculoskeletal symptoms including pain in back and muscles of the upper limbs [5]. The neuropsychological effects of on-call work include depressed mood [6], sleep deprivation and insomnia [7], sleepiness during the daytime [7, 8], reduced cognitive function [9, 10], and difficulty in concentrating [10]. Furthermore, a study on residents showed that long on-call work periods were associated with occupational injuries and malpractice [11].

On-call work may also affect socio-psychological stress. A previous study of Korean physicians showed an association between the frequency of on-call duty during the week and work-related stress [12]. Another study showed the relationship between on-call work and job dissatisfaction [2, 13] and also between on-call work and turnover intention [14], suggesting that job dissatisfaction from on-call work can affect turnover intention. In the US, the annual loss of operating budget due to job change is an estimated 5% [15]. Therefore, increased turnover of employees from on-call work may also become a social cost problem. Moreover, irregular working hours due to on-call work is associated with work-life imbalance [16], and a study of on-call workers and their spouses demonstrated the negative effects of on-call work on family life including constraints in their lives, forced sacrifice by spouses, and communication issues among family members [17]. These results clearly showed that on-call work affects not only work life but also life outside of work. In addition, the negative effects of on-call work on other family members were clearly demonstrated. Thus, on-call work not only has negative effects both physically and psychologically but may also result in socio-psychological stress, eventually affecting the quality of life of workers and their family members in addition to the social costs.

However, there are very few studies about on-call work in Korea. Moreover, previous studies generally focused on specific occupations (i.e., nurses and physicians) and no previous study on the effect of on-call work on health and other aspects has included a broad range of the job spectrum. This study aimed to investigate the possible associations between on-call work and its possible negative effects on health and other injuries by utilizing data from the 3rd Korean Working Conditions Survey performed in 2011 regarding on-call work-related personal and occupational characteristics and work environment in a large sample of workers representative of Korean workers nationwide.

## Methods

### Study population

This study was based on the 3rd Korean Working Conditions Survey performed by Korean Occupational Safety and Health Research Institute in 2011. The survey was conducted on a representative sample of workers (≥15 years). A worker was defined as “a person who during the reference week did any work for pay or profit”. A total of 50,032 subjects were included in the study, and in-person interviews were performed by professional interviewers from professional survey services after obtaining informed consent. This study focused on employed workers from the entire of 50,032 subjects. An employed worker was defined as “a person who signed either expressed or implied employment contract with individual, family, or business and is receiving salary or daily wage or spot goods in return for their labor”. Furthermore, since we aimed to investigate the effect of on-call work on the workers, only the employed workers who had been working for at least 1 year were included. Eventually, a total of 29,246 subjects were included in this study. Although the age limit for the Working Conditions Survey was 15 years, since the subjects included in this study had been working for at least 1 year, the subjects in this study were at least 16 years of age.

### On-call work

The subject was defined as working on-call if they responded “Yes” to the question “Do you work on-call (immediately providing work or service if contacted or called)?”

### Health problems and injuries

For health problems and injuries, the subject was defined as having health problems and injuries if they answered “Yes” to the question “Over the last 12 months, did you suffer from any of the following health problems?”. The questionnaire contained sub-categories for questions regarding health problems and injuries including hearing problems, skin problems, back pain, muscular pains in upper limbs, muscular pains in lower limbs, headaches or eyestrain, abdominal pain, respiratory difficulties, cardiovascular diseases, injuries, depression or anxiety, fatigue, insomnia and general sleep difficulties. Any report of hearing problems, skin problems, backache, muscular pains in upper limbs, muscular pains in lower limbs, headaches or eyestrain, abdominal pain, respiratory difficulties, cardiovascular diseases, fatigue was defined as a physical health problem. Similarly, if the subject had depression or anxiety, insomnia, general sleep difficulties, the subject was defined as having a psychological health problem.

### General and occupational characteristics

The general characteristics of the subjects investigated in this study included gender, age, education level, income level, alcohol consumption, smoking, obesity, and hypertension. For obesity and hypertension, the subject was defined as having a chronic condition if they answered “Yes” to the question “Were you ever diagnosed with chronic obesity or hypertension by your physician?”. However, for obesity, if the subject answered “Not obese” to the additional question “What is your current status in terms of obesity?”, the subject was not considered obese.

The occupational characteristics included job type, employment type, working hours, and shift work. Two methods were used to categorize the job types. First, the subjects were asked, “What is the job type that best describes your current occupation?”. Based on their responses, professional workers and senior managers were defined as “Professional”, general office workers as “Office”, sales workers and service providers as “Service”, and skilled/semi-skilled/unskilled occupations and agricultural/forestry/fishery workers as “Physical”. Second, based on the middle categorization of the Korean Employment Classification of Occupation (KECO), the subjects’ occupations were categorized into 24 job groups. Employment type was defined as regular or temporary, and working hours were categorized as ≤ 40 h, 41 – 60 h, or > 60 h per week.

### Work environment and work-related stress

Work environment was assessed in terms of exposures to physical, chemical, and ergonomic factors. If the subject answered “All of the time”, “Almost all of the time”, “Around 3/4 of the time”, “Around half of the time” or “Around 1/4 of the time” to the question “Are you exposed at work to ...?”, the subject was considered to be exposed to the factor. However, if the subject answered “Almost never” or “Never” to the same question, the subject was not considered to be exposed to the factor. The physical factors included the vibrations, noise, high temperatures, and low temperatures. The chemical factors included breathing in smoke, fumes, powder or dust, vapors such as solvents and thinners, handling or being in skin contact with chemical products or substances, and tobacco smoke from other people. Lastly, the ergonomic factors included tiring or painful positions, lifting or moving people, carrying or moving heavy loads, standing, and repetitive hand or arm movements.

For work-related stress, if the subject answered “Always”, “Most of the time”, or “Sometimes” to the question “For each of the following statements, please select the response which best describes your work situation – You experience stress in your work.”, the subject was defined as having work-related stress. If the answer was “Rarely” or “Never”, the subject was defined as being free of work-related stress.

### Data analysis

This study utilized IBM SPSS Statistics for Windows version 19.0 (IBM Corp: Armonk, NY, USA) for analysis of the data after applying weighting adjustments. Personal and occupational characteristics, work environment, and work-related stress were analysed using frequency analysis. Chi-square analysis was used to determine the associations between on-call work and characteristics of the subject and on-call work and health problems and injuries. In order to determine the risk of health problems and injuries due to on-call work, bivariate logistic regression analysis was performed to calculate the odds ratios. Model I was the analysis after adjusting for personal characteristics, while Model II was additionally adjusted for occupational characteristics including job type, employment type, working hours, and shift work. Model III further adjusted for physical, chemical, and ergonomic factors of the work environment, while Model IV had additional adjustment for work-related stress in addition to all controls from Model III.

## Results

### General and occupational characteristics of the study participants

A total of 29,246 subjects were included in this study. Frequency analysis and descriptive statistics were used to analyze the personal and occupational characteristics, work environment, and work-related stress. The personal characteristics of the subjects included the following: 60.9% of the subjects were male; 59.1% were between 30 and 49 years of age; 55.7% had education levels higher than college graduation; 53.5% were non-smokers; 51.5% drank alcohol once a week or less; 98.2% were not obese; and 95.3% were not diagnosed with hypertension. A majority of the subjects were “physical” workers (33.6%), and only 8.7% were “professional” workers. Moreover, a majority of the subjects were regular employees, and 51.5% worked between 41 and 60 h per week, while 39.6% worked 40 h or less per week. Most of the subjects were not working on a rotating basis (i.e., no shift work). More than half of the subjects responded that they did not have exposure to physical, chemical, or ergonomic factors in their work environment, but 72.1% reported that they suffered from work-related stress.

### Difference of general occupational characteristics according to on-call work

2722 subjects (9.3%) were on-call workers; the remaining 26,524 (90.7%) were not on-call workers. Chi-square analysis was used to analyze the differences in each factor according to the on-call work status. The results revealed significant differences between on-call workers and non-on-call workers in all factors except for hypertension and working hours. Assessment of the general characteristics revealed a higher rate of on-call workers in the following groups: male, ≥50 years, high school graduates, monthly income ≥2 million won, smokers, and obese subjects. Among job types, there were more on-call workers in the “physical” group, while the “professional” group had fewer on-call workers. The rate of on-call workers was also higher for temporary and shift workers. Assessment of work environment revealed a higher rate of on-call workers exposed to physical, chemical, or ergonomic factors, as well as work-related stress (Table 1).

**Table 1:** Comparisons of the characteristics of the study subjects according to the on-call work

Characteristics		On-call work n(%)		*P*-value
		Yes	No	
		2722(9.3)	26,524(90.7)	
Sex	Male	1813(10.2)	15,999(89.8)	< 0.001
	Female	909(7.9)	10,525(92.1)	
Age(years)	15-29	417(8.2)	4684(91.8)	0.004
	30-49	1621(9.4)	15,651(90.6)	
	≥ 50	684(10.0)	6189(90.0)	
Education	Below middle school	261(9.5)	2489(90.5)	0.008
	High school	1018(10.0)	9174(90.0)	
	College and beyond	1443(8.9)	14,861(91.1)	
Income	< 100	156(6.7)	2183(93.3)	< 0.001
(10,000won/month)	100-199	948(8.7)	9962(91.3)	
	200-299	945(10.4)	8161(89.6)	
	≥ 300	673(9.8)	6208(90.2)	
Smoking	Never	1376(8.8)	14,263(91.2)	< 0.001
	Quit	273(8.5)	2957(91.5)	
	Current	1073(10.3)	9304(89.7)	
Drinking	No	608(9.8)	5576(90.2)	0.004
	≤ 1/week	1318(8.8)	13,732(91.2)	
	≥ 2/week	796(9.9)	7216(90.1)	
Obesity	No	2658(9.3)	26,063(90.7)	0.022
	Yes	64(12.2)	461(87.8)	
Hypertension	No	2597(9.3)	25,278(90.7)	0.794
	Yes	125(9.1)	1246(90.9)	
Employment type	Regular	2266(9.1)	22,651(90.9)	0.003
	Temporary	456(10.5)	3873(89.5)	
Working time	≤ 40	1034(8.9)	10,553(91.1)	0.159
(Hours/week)	41-60	1432(9.5)	13,631(90.5)	
	> 60	256(9.9)	2340(90.1)	
Job type	Professional	185(7.3)	2356(92.7)	< 0.001
	Office	860(9.1)	8607(90.9)	
	Service	677(9.1)	6749(90.9)	
	Physical	1000(10.2)	8812(89.8)	
Shift work	No	2200(8.3)	24,247(91.7)	< 0.001
	Yes	522(18.6)	2277(81.4)	
Physical exposure	No	1580(8.5)	16,953(91.5)	< 0.001
	Yes	1142(10.7)	9571(89.3)	
Chemical exposure	No	1719(8.1)	19,563(91.9)	< 0.001
	Yes	1003(12.6)	6961(87.4)	
Ergonomic exposure	No	2199(9.0)	22,285(91.0)	< 0.001
	Yes	523(11.0)	4239(89.0)	
Work-related stress	No	673(8.3)	7481(91.7)	< 0.001
	Yes	2049(9.7)	19,043(90.3)	

The subjects were divided into 24 job classifications based on KECO categorizations and were analyzed for on-call work. The highest rate of on-call workers (31.7%) were those in the law, police, firefighting, and prison-related job group. The job classifications that had higher rates of on-call workers than healthcare and medical-related jobs (12.8%) include soldiers (30.2%), driver and transportation-related jobs (14.6%), information and communications-related jobs (13.4%), and construction-related jobs (12.9%). Moreover, although the proportions of on-call workers were low for management, accounting, and office-related (8.7%) and sales (8.1%) jobs, the absolute number of on-call workers in these job classifications were quite high, at 21.2%(578 subjects) and 7.8%(214 subjects) of the entire on-call workers, respectively (Table 2).

**Table 2:** Comparisons of the on-call work of the study subjects according to the job classification

Job classification (KECO^a^)	On-call work n(%)	
	Yes	No
	2722(9.3)	26,524(90.7)
Law, Police, Firefighting, Prison	117(31.7)	252(68.3)
Soldier	26(30.2)	60(69.8)
Drivers and transportation	243(14.6)	1412(85.4)
Information and Communications	74(13.4)	478(86.6)
Construction	241(12.9)	1625(87.1)
Public healthcare, Medical	206(12.8)	1406(87.2)
Management	68(11.2)	538(88.8)
Agriculture, Forestry, and Fishing	17(10.5)	145(89.5)
Security and Janitorial staff and cleaning	166(9.6)	1563(90.4)
Electricity and Electronics	96(9.4)	927(90.6)
Culture, Art, Design, Broadcasting	53(9.3)	517(90.7)
Management, Accounting, Office job	579(8.6)	6181(91.4)
Machine	129(8.4)	1404(91.6)
Sales	214(8.1)	2444(91.9)
Finance and Insurance	80(7.6)	967(92.4)
Environment, Printing, Woodworking, Furniture, crafts, simple manufacturing	79(7.6)	959(92.4)
Food processing	15(7.1)	197(92.9)
Social welfare and Religion	41(6.8)	562(93.2)
Food service	90(6.6)	1265(93.4)
Chemistry	16(6.6)	228(93.4)
Beauty, Accommodations, Tourism, Entertainment, and Sports	50(6.3)	741(93.7)
Fabrics and Clothing	23(5.6)	390(94.4)
Materials (Metal, Glass, Clay, Cement)	26(5.4)	455(94.6)
Research (Education, Natural/Social Sciences)	73(3.9)	1808(96.1)

^a^*KECO* Korean Employment Classification of Occupation

### On-call work and its association with health problems, injuries

Chi-square analysis was performed to determine the relationship between on-call work and health problems and injuries. The results indicated that all health problems except for cardiovascular disease had statistically significant higher prevalence among on-call workers (Table 3).

**Table 3:** Comparisons of the health problems and injuries of the study subjects according to the on-call work

Health problems and injuries		On-call work n(%)		*P*-value
		Yes	No	
		2722(9.3)	26,524(90.7)	
Physical health problems	Yes	1540(10.6)	13,051(89.4)	< 0.001
	No	1182(8.1)	13,473(91.9)	
Hearing problems	Yes	92(18.8)	397(81.2)	< 0.001
	No	2630(9.1)	26,127(90.9)	
Skin problems	Yes	108(15.3)	598(84.7)	< 0.001
	No	2614(9.2)	25,926(90.8)	
Back pain	Yes	368(11.9)	2723(88.1)	< 0.001
	No	2354(9.0)	23,801(91.0)	
Muscular pains in upper limbs	Yes	927(10.5)	7905(89.5)	< 0.001
	No	1795(8.8)	18,619(91.2)	
Muscular pains in lower limbs	Yes	609(10.8)	5008(89.2)	< 0.001
	No	2113(8.9)	21,516(91.1)	
Headache, eyestrain	Yes	661(12.4)	4672(87.6)	< 0.001
	No	2061(8.6)	21,852(91.4)	
Abdominal pain	Yes	53(12.8)	361(87.2)	0.014
	No	2669(9.3)	26,163(90.7)	
Respiratory difficulties	Yes	27(14.1)	164(85.9)	0.021
	No	2695(9.3)	26,360(90.7)	
Cardiovascular diseases	Yes	28(8.5)	300(91.5)	0.629
	No	2694(9.3)	26,224(90.7)	
Fatigue	Yes	765(11.9)	5680(88.1)	< 0.001
	No	1957(8.6)	20,844(91.4)	
Psychological health problems	Yes	128(13.1)	847(86.9)	< 0.001
	No	2594(9.2)	25,677(90.8)	
Depression or anxiety	Yes	54(13.0)	360(87.0)	0.008
	No	2668(9.3)	26,164(90.7)	
Insomnia and sleep difficulties	Yes	102(14.4)	608(85.6)	< 0.001
	No	2620(9.2)	25,916(90.8)	
Injuries	Yes	144(24.2)	457(75.8)	< 0.001
	No	2578(9.0)	26,067(91.0)	

Bivariate logistic regression analysis was performed to determine the effect of on-call work on the risk of health problems and injuries. The risks were higher for on-call workers than those for non-on-call workers for the majority of health problems and injuries. This outcome did not change even after adjusting for personal and occupational characteristics, work environment, and work-related stress. The odds ratios for on-call workers in terms of physical health problems, psychological health problems, and injuries were 1.33 (95% confidence interval [CI] 1.22-1.44), 1.31 (95% CI 1.08-1.60), and 2.76 (95% CI 2.26-3.37), respectively. Analysis of the detailed symptoms revealed odds ratios in on-call workers of 2.06 for hearing problems (95% CI 1.63-2.62); 1.71 for skin problems (95% CI 1.38-2.12); 1.22 for back pain (95% CI 1.08-1.38); 1.23 for muscular pains in upper limbs (95% CI 1.12-1.34); 1.27 for muscular pains in lower limbs (95% CI 1.15-1.40); 1.46 for headache, eye fatigue (95% CI 1.32-1.60); 1.37 for abdominal pain (95% CI 1.02-1.85); 1.43 for depression or anxiety disorders (95% CI 1.07-1.93); 1.36 for fatigue (95% CI 1.24-1.49); and 1.41 for insomnia and general sleep difficulties (95% CI 1.13-1.76). However, there were no statistically significant differences between groups for the risks of respiratory difficulties or cardiovascular diseases (Table 4).

**Table 4:** Odds ratios of health problems and injuries on on-call work from logistic regression models

	Model I	Model II	Model III	Model IV
	OR^a^ (95%CI^b^)	OR(95%CI)	OR(95%CI)	OR(95%CI)
Physical health problems	1.35(1.25-1.47)	1.35(1.25-1.47)	1.34(1.23-1.45)	1.33(1.22-1.44)
Hearing problems	2.18(1.73-2.75)	2.20(1.74-2.78)	2.07(1.63-2.63)	2.06(1.63-2.62)
Skin problems	1.80(1.46-2.23)	1.83(1.48-2.26)	1.72(1.39-2.13)	1.71(1.38-2.12)
Back pain	1.25(1.11-1.41)	1.22(1.09-1.38)	1.22(1.08-1.38)	1.22(1.08-1.38)
Muscular pains in upper limbs	1.22(1.12-1.33)	1.23(1.13-1.34)	1.23(1.13-1.34)	1.23(1.12-1.34)
Muscular pains in lower limbs	1.26(1.14-1.39)	1.27(1.15-1.40)	1.27(1.15-1.41)	1.27(1.15-1.40)
Headache, eyestrain	1.50(1.36-1.64)	1.50(1.36-1.65)	1.47(1.33-1.61)	1.46(1.32-1.31)
Abdominal pain	1.44(1.07-1.93)	1.47(1.09-1.98)	1.37(1.02-1.85)	1.37(1.02-1.85)
Respiratory difficulties	1.59(1.05-2.39)	1.51(1.00-2.29)	1.38(0.91-2.10)	1.39(0.91-2.11)
Cardiovascular diseases	0.93(0.63-1.39)	0.91(0.61-1.36)	0.89(0.60-1.34)	0.90(0.60-1.34)
Fatigue	1.42(1.30-1.55)	1.39(1.27-1.52)	1.37(1.25-1.50)	1.36(1.24-1.49)
Psychological health problems	1.52(1.26-1.85)	1.38(1.13-1.67)	1.32(1.08-1.61)	1.31(1.08-1.60)
Depression or anxiety	1.50(1.12-2.00)	1.49(1.11-2.00)	1.44(1.07-1.93)	1.43(1.07-1.93)
Insomnia and sleep difficulties	1.68(1.35-2.08)	1.46(1.17-1.82)	1.41(1.13-1.77)	1.41(1.13-1.76)
Injuries	3.00(2.47-3.65)	3.07(2.52-3.74)	2.76(2.26-3.37)	2.76(2.26-3.37)

Model I: Adjusted for personal characteristics (gender, age group, education, income, smoking, drinking, obesity, hypertension)

Model II: Model I + Adjusted for occupational characteristics (job type, employment type, working hours, shift work)

Model III: Model II + Adjusted for working environment (physical, chemical, ergonomic exposure)

Model IV: Model III + Adjusted for work-related stress

^a^*OR* Odds ratio

^b^*CI* Confidence interval

## Discussion

This study utilized data from the 3rd Korean Working Conditions Survey on employed workers and analysed the relationships between their characteristics and health problems and injuries as well as the impact of on-call work on health problems and injuries.

Analysis of the subjects’ characteristics according to the on-call work showed differences in both personal characteristics – gender, age, education, income, smoking, alcohol consumption, and obesity – and occupational characteristics – job type, employment type, and shift work. Especially for the employment type, temporary workers were more often on-call workers. Unstable employment status in these temporary workers likely forces them to cope with stressful work environments. Moreover, previous studies reported that on-call workers have extended working hours (both daytime and nighttime) [1] and are exposed to both shift and night-time work [14], which may explain why on-call workers feel more fatigued in general. In fact, the analysis findings of the current study revealed a higher rate of on-call workers for shift work. However, there was no statistically significant difference in the rate of on-call workers based on working hours. On-call workers had increased exposure to all analysed physical, chemical, and ergonomic factors, suggesting that these workers tend to work in worse work environments. The rate of on-call workers was also higher in the group that experienced work-related stress, further supporting the previous finding that on-call work is a key factor of work-related stress [2–4]. The rates of on-call workers were higher in law, police, firefighting, and prison-related jobs; soldiers; driving and transportation-related jobs; information and communications-related jobs; and construction-related jobs, compared to the rate in healthcare and medical-related jobs, which was traditionally thought as a job category with a high rate of on-call workers. This result indicates that in addition to traditional studies of on-call workers that focus on healthcare and medical-related jobs, additional studies are necessary in order to analyse other job groups.

Analysis of the effects of on-call work on health problems and injuries showed higher odds ratios for the majority of health-related issues and injuries in on-call workers compared to those of non-on-call workers, with exceptions of respiratory difficulties, cardiovascular diseases. These results did not change even after adjusting for personal and occupational characteristics, work environment, and work-related stress.

To our knowledge, no previous study has investigated the relationship between on-call work and hearing problems, skin problems, or abdominal pain. A potential explanation that could explain the relevance of on-call work with these problems is circadian disruption. In a study on sudden sensorineural hearing loss patients, 61.8% of the subjects reported insomnia before suffering from hearing loss, and the circadian clock gene was reduced compared to the control group [18]. It is thought that circadian disruption can be caused by irregular sleeping patterns that may occur during on-call work, which may cause hearing problems. Previous study have shown that exposure to light at night causes circadian disruption and decreased melatonin synthesis [19]. Melatonin may protect against psoriasis because it regulates the inflammatory response and antioxidant activity [20]. Previous study on shift workers have also shown that circadian disruption and decreased melatonin are associated with psoriasis [21]. In addition, in a mouse-based experiment, it was possible to regulate the circadian clock to control psoriasis-like skin inflammation by controlling the IL-23 expression in T cells [22], suggesting that circadian disruption may be related to inflammatory skin problems. Our study shows that the risk of skin problems is higher for on-call workers even after adjusting for the shift work. This may due to on-call workers also have disrupted circadian rhythm, which may caused by their irregular working hours, and light exposure at night. Previous studies have demonstrated the relationship between on-call work and indigestion [4] and possible induction of gastrointestinal tract diseases due to an imbalanced lifestyle from irregular eating habits and lack of sleep [23]. Thus, on-call workers who have irregular eating habits – since they are expected to work when needed – were expected to have various gastrointestinal tract-related issues and diseases. However, similar to hearing problems, additional studies are necessary in order to identify the potential mediating factors of skin problems and abdominal pain. However this is only one potential explanation that could explain the relevance of on-call work with hearing problems, skin problems and abdominal pain, so further studies are required.

A previous study reported that on-call work is associated with musculoskeletal pain (i.e., back and shoulder pain) [5], a result in agreement with our findings that on-call work is associated with back and upper/lower limb muscle pain. Furthermore, one possible explanation for these musculoskeletal symptoms was a lack of a rest and recovery period [24]. On-call workers have a shorter time for rest and recovery due to irregular working hours, which could lead to musculoskeletal symptoms in these workers.

A previous study of Finnish anesthesiologists reported that on-call work is associated with exhaustion, frustration, sleep disorders, memory disorders, and headache. Greater work-related burdens from on-call work resulted in an increased severity of these symptoms, and these symptoms disappeared during vacation [4]. These results indicate a strong relationship between on-call work and symptoms such as headache or fatigue. From our study of Korean workers, on-call work was associated with headache, eye fatigue, and general fatigue.

A previous study discussed depressed mood in on-call workers [6], and another suggested the possible relationship between on-call work and negative emotions [25]. Furthermore, on-call work was associated with daytime sleepiness, insomnia, and sleep deprivation [7, 8], and possibly dysthymic disorder from continued sleep deprivation [26]. These results suggest that on-call work may be a cause of sleep deprivation and sleep disorders as well as for negative emotions in on-call workers who are constantly sleep deprived due to the nature of their work. Moreover, previous studies showed the association between on-call work and work dissatisfaction [2, 13] and the result of a study on the relationship between work dissatisfaction and general mental health (i.e., depression and anxiety) [27] indicated that work dissatisfaction from on-call work might cause depression or anxiety. These results were further supported by the findings of this study showing that on-call work increases the risks of depression or anxiety disorder, insomnia, or sleep disorders.

A previous study suggested the association between on-call work and work-related injuries [11]; the present study also found this association in Korean workers. On-call work is associated with reduced cognitive function and concentration [9, 10] and studies have shown that sleep deprivation can cause reduced cognitive function [28, 29]. Therefore, reduced cognitive function and concentration from on-call work can increase the risk of injuries.

This study has many strengths. To our knowledge, it is the first Korean study to determine the association between on-call work and health problems and injuries. Furthermore, unlike traditional studies that focused on a specific job group, this study included a wide range of job groups in order to investigate the relationship between on-call work and health problems, injuries. In the present study, higher rates of on-call workers were found in other job groups compared to that of the healthcare-related job group, which was the target job group in previous studies. This finding suggests the need for future studies focusing on other job groups. Second, this study utilized data from the Korean Working Conditions Survey and the 29,246 subjects included in this study were representative of Korean employed workers nationwide. In addition, the utilization of trained investigators to perform the survey minimized arbitrary interpretation of the survey responses.

This study had several limitations. First, the cross-sectional study design based on data from the Korean Working Conditions Survey made it difficult to identify the clear causal relationship between on-call work and health problems and injuries. Future studies are needed to identify the clear causal relationships. Second, the only measurement tools for variables were the responses to the Korean Working Conditions Survey. More specifically, for the work environment, exposures were determined by dichotomous measures of the survey responses rather than actual assessments of work the environments. Furthermore, instead of objective measurements such as physician interviews, blood test, or medical imaging, self-reporting was used to assess health problems. And there may be a misclassification of the job types, because there were only four job types, so jobs of different character may belong to same category. Lastly, the assessed medical history of the subjects included only the presence of obesity and hypertension.

## Conclusion

This is one of the first studies identified a significant relationship between on-call work in Korean workers and health problems and injuries. Moreover, this study included a wide range of job groups in order to investigate the relationship between on-call work and health problems, injuries. Future studies are required to identify clear causal relationships; similarly, additional discussions are needed in order to reduce the adverse effects of on-call work.

## Abbreviations

EWCS: European working conditions survey
KECO: Korean employment classification of occupation

## References

[1] Akerstedt T (2008). Altered sleep/wake patterns and mental performance. Physiol Behav.

[2] Dowell A, Hamilton S (2000). Job satisfaction, psychological morbidity, and job stress among New Zealand general practitioners. N Z Med J.

[3] Chong A, Killeen O, Clarke T (2004). Work-related stress among paediatric non-consultant hospital doctors. Ir Med J.

[4] Lindfors PM, Nurmi KE, Meretoja OA (2006). On call stress among Finnish anaesthetists. Anaesthesia.

[5] Alison MT, Rong L, Jeanne GB, Jane L, Gary L (2006). Longitudinal relationship of work hours, mandatory overtime, and on-call to musculoskeletal problems in nurses. Am J Ind Med.

[6] Rankin HJ, Serieys NM, Elliott-Binns CP (1987). Determinants of mood in general practitioners. Br Med J (Clin Res Ed).

[7] Kaneita Y, Ohida T (2011). Association of current work and sleep situations with excessive daytime sleepiness and medical incidents among Japanese physicians. J Clin Sleep Med.

[8] Wali SO, Qutah K, Abushanab L, Basamh R, Abushanab J, Krayem A (2013). Effect of on-call-related sleep deprivation on physicians’ mood and alertness. Ann Thorac Med.

[9] Rubin R, Orris P, Lau SL, Hryhorczuk DO, Furner S, Letz R (1991). Neurobehavioral effects of the on-call experience in housestaff physicians. J Occup Med.

[10] Florian E, Markus R, Heinz Z (2014). Effects of 24h working on-call on psychoneuroendocrine and oculomotor function: a randomized cross-over trial. Psychoneuroendocrinology.

[11] Lockley SW, Barger LK, Ayas NT (2007). Effects of health care provider work hours and sleep deprivation on safety and performance. Jt Comm J Qual Patient Saf.

[12] Kang YS, Kam S, Lee SW, Chun BY, Yeh MH (2001). Job stress and its related factors in south Korean doctors. J Prev Med Public Health.

[13] Van HI, Verhoeven AA, Groenier KH, Groothoff JW, De HJ (2006). Job satisfaction among general practitioners: a systematic literature review. Eur J Gen Pract.

[14] Heponiemi T, Kouvonen A, Vanska J (2008). Effects of active on-call hours on physicians’ turnover intentions and well-being. Scand J Work Environ Health.

[15] Waldman JD, Kelly F, Aurora S, Smith HL (2004). The shocking cost of turnover in health care. Health Care Manag Rev.

[16] Yildirim D, Aycan Z (2008). Nurses' work demands and work-family conflict: a questionnaire survey. Int J Nurs Stud.

[17] Emmett BM, Dovey SM, Wheeler BJ (2013). After-hours on-call: the effect on paediatricians’ spouses and families. J Paediatr Child Health.

[18] Yang CH, Hwang CF, Lin PM, Chuang JH, Hsu CM, Lin SF, Yang MY (2015). Sleep disturbance and altered expression of circadian clock genes in patients with sudden sensorineural hearing loss. Medicine.

[19] Scheer FA, Hilton MF, Mantzoros CS, Shea SA (2009). Adverse metabolic and cardiovascular consequences of circadian misalignment. Proc Natl Acad Sci U S A.

[20] Bonnefont-Rousselot D, Collin F (2010). Melatonin: action as antioxidant and potential applications in human disease and aging. Toxicology.

[21] Li WQ, Qureshi AA, Schernhammer ES, Han J (2013). Rotating night-shift work and risk of psoriasis in US women. J Invest Dermatol.

[22] Ando N, Nakamura Y, Aoki R, Ishimaru K, Ogawa H, Okumura K, Shibata S, Shimada S, Nakao A (2015). Circadian gene clock regulates psoriasis-like skin inflammation in mice. J Invest Dermatol.

[23] Konturek PC, Brzozowski T, Konturek SJ (2011). Gut clock: implication of circadian rhythms in the gastointestinal tract. J Physiol Pharmacol.

[24] Nicole J, IJmert K, Ludovic VA, Frans N, Piet VDB (2003). Need for recovery from work: evaluating short-term effects of working. Ergonomics.

[25] Rose M, Manser T, Ware JC (2008). Effects of call on sleep and mood in internal medicine residents. Behav Sleep Med.

[26] Birchler-Pedross A, Schroder CM, Munch M (2009). Subjective well-being is modulated by circadian phase, sleep pressure, age, and gender. J Biol Rhythm.

[27] Faragher EB, Cass M, Cooper CL (2005). The relationship between job satisfaction and health: a meta-analysis. Occup Environ Med.

[28] Wertz AT, Ronda JM, Czeisler CA, WrightJr KP (2006). Effects of sleep inertia on cognition. J Am Med Assoc.

[29] Goel N, Rao H, Durmer JS, Dinges DF (2009). Neurocognitive consequences of sleep deprivation. Semin Neurol.

